# Supplementary material for: Resolving the full spectrum of human genome variation using Linked-Reads
Source: Genome Res. 2019 Apr;29(4):635–45. doi: 10.1101/gr.234443.118 (PMC6442396; doi:10.1101/gr.234443.118)
Supplement: Supplemental Material [file supp_gr.234443.118_Supplemental_longranger-2.2.zip › longranger-2.2.tar.bz2!/lr2.2-public/lib/stan/cmdstan/stan/doc/api/html/statement__grammar_8hpp_source.html]

Stan: src/stan/gm/grammars/statement\_grammar.hpp Source File


|  |  |
| --- | --- |
|  | Stan  2.5.0  probability, sampling & optimization |


- Main Page
- Related Pages
- Namespaces
- Classes
- Files

- File List
- File Members

All Classes Namespaces Files Functions Variables Typedefs Enumerations Enumerator Friends Macros Pages

- src
- stan
- gm
- grammars

statement\_grammar.hpp

Go to the documentation of this file.

1 #ifndef STAN\_\_GM\_\_PARSER\_\_STATEMENT\_GRAMMAR\_\_HPP

2 #define STAN\_\_GM\_\_PARSER\_\_STATEMENT\_GRAMMAR\_\_HPP

3

4 #include <string>

5 #include <sstream>

6 #include <vector>

7

8 #include <boost/spirit/include/qi.hpp>

9

10 #include <stan/gm/ast.hpp>

11 #include <stan/gm/grammars/whitespace\_grammar.hpp>

12 #include <stan/gm/grammars/expression\_grammar.hpp>

13 #include <stan/gm/grammars/var\_decls\_grammar.hpp>

14 #include <stan/gm/grammars/statement\_2\_grammar.hpp>

15

16 namespace stan {

17

18  namespace gm {

19

20  template <typename Iterator>

21  struct statement\_grammar

22  : boost::spirit::qi::grammar<Iterator,

23  statement(bool,var\_origin,bool),

24  whitespace\_grammar<Iterator> > {

25

26

27

28  statement\_grammar(variable\_map& var\_map,

29  std::stringstream& error\_msgs);

30

31

32  // global info for parses

33  variable\_map& var\_map\_;

34  std::stringstream& error\_msgs\_;

35

36  // grammars

37  expression\_grammar<Iterator> expression\_g;

38  var\_decls\_grammar<Iterator> var\_decls\_g;

39  statement\_2\_grammar<Iterator> statement\_2\_g;

40

41  // rules

42  boost::spirit::qi::rule<Iterator,

43  assignment(var\_origin),

44  whitespace\_grammar<Iterator> >

45  assignment\_r;

46

47  boost::spirit::qi::rule<Iterator,

48  expression(var\_origin),

49  whitespace\_grammar<Iterator> >

50  non\_lvalue\_assign\_r;

51

52

53  boost::spirit::qi::rule<Iterator,

54  std::vector<expression>(var\_origin),

55  whitespace\_grammar<Iterator> >

56  dims\_r;

57

58

59  boost::spirit::qi::rule<Iterator,

60  distribution(var\_origin),

61  whitespace\_grammar<Iterator> >

62  distribution\_r;

63

64

65  boost::spirit::qi::rule<Iterator,

66  increment\_log\_prob\_statement(bool,var\_origin),

67  whitespace\_grammar<Iterator> >

68  increment\_log\_prob\_statement\_r;

69

70  boost::spirit::qi::rule<Iterator,

71  boost::spirit::qi::locals<std::string>,

72  for\_statement(bool,var\_origin,bool),

73  whitespace\_grammar<Iterator> >

74  for\_statement\_r;

75

76

77  boost::spirit::qi::rule<Iterator,

78  while\_statement(bool,var\_origin,bool),

79  whitespace\_grammar<Iterator> >

80  while\_statement\_r;

81

82

83  boost::spirit::qi::rule<Iterator,

84  print\_statement(var\_origin),

85  whitespace\_grammar<Iterator> >

86  print\_statement\_r;

87

88  boost::spirit::qi::rule<Iterator,

89  reject\_statement(var\_origin),

90  whitespace\_grammar<Iterator> >

91  reject\_statement\_r;

92

93

94  boost::spirit::qi::rule<Iterator,

95  return\_statement(var\_origin),

96  whitespace\_grammar<Iterator> >

97  return\_statement\_r;

98

99  boost::spirit::qi::rule<Iterator,

100  return\_statement(var\_origin),

101  whitespace\_grammar<Iterator> >

102  void\_return\_statement\_r;

103

104

105

106

107  boost::spirit::qi::rule<Iterator,

108  printable(var\_origin),

109  whitespace\_grammar<Iterator> >

110  printable\_r;

111

112  boost::spirit::qi::rule<Iterator,

113  std::string(),

114  whitespace\_grammar<Iterator> >

115  printable\_string\_r;

116

117

118  boost::spirit::qi::rule<Iterator,

119  std::string(),

120  whitespace\_grammar<Iterator> >

121  identifier\_r;

122

123  boost::spirit::qi::rule<Iterator,

124  std::vector<var\_decl>(),

125  whitespace\_grammar<Iterator> >

126  local\_var\_decls\_r;

127

128  boost::spirit::qi::rule<Iterator,

129  no\_op\_statement(),

130  whitespace\_grammar<Iterator> >

131  no\_op\_statement\_r;

132

133  boost::spirit::qi::rule<Iterator,

134  std::vector<expression>(var\_origin),

135  whitespace\_grammar<Iterator> >

136  opt\_dims\_r;

137

138  boost::spirit::qi::rule<Iterator,

139  range(var\_origin),

140  whitespace\_grammar<Iterator> >

141  range\_r;

142

143  boost::spirit::qi::rule<Iterator,

144  sample(bool,var\_origin),

145  whitespace\_grammar<Iterator> >

146  sample\_r;

147

148  boost::spirit::qi::rule<Iterator,

149  statement(bool,var\_origin,bool),

150  whitespace\_grammar<Iterator> >

151  statement\_r;

152

153  boost::spirit::qi::rule<Iterator,

154  boost::spirit::qi::locals<std::vector<var\_decl> >,

155  statements(bool,var\_origin,bool),

156  whitespace\_grammar<Iterator> >

157  statement\_seq\_r;

158

159  boost::spirit::qi::rule<Iterator,

160  range(var\_origin),

161  whitespace\_grammar<Iterator> >

162  truncation\_range\_r;

163

164  boost::spirit::qi::rule<Iterator,

165  variable\_dims(var\_origin),

166  whitespace\_grammar<Iterator> >

167  var\_lhs\_r;

168

169  };

170

171

172  }

173 }

174

175 #endif

stan::gm::variable\_dims

**Definition:** ast.hpp:288

stan::gm::statement

**Definition:** ast.hpp:634

stan::gm::statement\_grammar::printable\_r

boost::spirit::qi::rule< Iterator, printable(var\_origin), whitespace\_grammar< Iterator > > printable\_r

**Definition:** statement\_grammar.hpp:110

stan::gm::statement\_grammar::range\_r

boost::spirit::qi::rule< Iterator, range(var\_origin), whitespace\_grammar< Iterator > > range\_r

**Definition:** statement\_grammar.hpp:141

stan::gm::distribution

**Definition:** ast.hpp:194

stan::gm::statement\_2\_grammar

**Definition:** statement\_2\_grammar.hpp:22

stan::gm::statement\_grammar::for\_statement\_r

boost::spirit::qi::rule< Iterator, boost::spirit::qi::locals< std::string >, for\_statement(bool, var\_origin, bool), whitespace\_grammar< Iterator > > for\_statement\_r

**Definition:** statement\_grammar.hpp:74

stan::gm::statements

**Definition:** ast.hpp:185

stan::gm::range

**Definition:** ast.hpp:401

stan::gm::assignment

**Definition:** ast.hpp:828

expression\_grammar.hpp

stan::gm::statement\_grammar::local\_var\_decls\_r

boost::spirit::qi::rule< Iterator, std::vector< var\_decl >), whitespace\_grammar< Iterator > > local\_var\_decls\_r

**Definition:** statement\_grammar.hpp:126

stan::gm::expression\_grammar

**Definition:** expression07\_grammar.hpp:23

stan::gm::statement\_grammar::identifier\_r

boost::spirit::qi::rule< Iterator, std::string(), whitespace\_grammar< Iterator > > identifier\_r

**Definition:** statement\_grammar.hpp:121

stan::gm::no\_op\_statement

**Definition:** ast.hpp:763

stan::gm::statement\_grammar::reject\_statement\_r

boost::spirit::qi::rule< Iterator, reject\_statement(var\_origin), whitespace\_grammar< Iterator > > reject\_statement\_r

**Definition:** statement\_grammar.hpp:91

stan::gm::statement\_grammar::truncation\_range\_r

boost::spirit::qi::rule< Iterator, range(var\_origin), whitespace\_grammar< Iterator > > truncation\_range\_r

**Definition:** statement\_grammar.hpp:162

stan::gm::statement\_grammar::printable\_string\_r

boost::spirit::qi::rule< Iterator, std::string(), whitespace\_grammar< Iterator > > printable\_string\_r

**Definition:** statement\_grammar.hpp:115

stan::gm::var\_decls\_grammar

**Definition:** var\_decls\_grammar.hpp:20

stan::gm::statement\_grammar::statement\_2\_g

statement\_2\_grammar< Iterator > statement\_2\_g

**Definition:** statement\_grammar.hpp:39

stan::gm::statement\_grammar::statement\_r

boost::spirit::qi::rule< Iterator, statement(bool, var\_origin, bool), whitespace\_grammar< Iterator > > statement\_r

**Definition:** statement\_grammar.hpp:151

stan::gm::for\_statement

**Definition:** ast.hpp:718

stan::gm::increment\_log\_prob\_statement

**Definition:** ast.hpp:712

stan::gm::printable

**Definition:** ast.hpp:256

statement\_2\_grammar.hpp

stan::gm::print\_statement

**Definition:** ast.hpp:745

stan::gm::statement\_grammar::statement\_seq\_r

boost::spirit::qi::rule< Iterator, boost::spirit::qi::locals< std::vector< var\_decl > >, statements(bool, var\_origin, bool), whitespace\_grammar< Iterator > > statement\_seq\_r

**Definition:** statement\_grammar.hpp:157

stan::gm::statement\_grammar::error\_msgs\_

std::stringstream & error\_msgs\_

**Definition:** statement\_grammar.hpp:34

stan::gm::statement\_grammar::print\_statement\_r

boost::spirit::qi::rule< Iterator, print\_statement(var\_origin), whitespace\_grammar< Iterator > > print\_statement\_r

**Definition:** statement\_grammar.hpp:86

stan::gm::variable\_map

**Definition:** ast.hpp:439

stan::gm::statement\_grammar::while\_statement\_r

boost::spirit::qi::rule< Iterator, while\_statement(bool, var\_origin, bool), whitespace\_grammar< Iterator > > while\_statement\_r

**Definition:** statement\_grammar.hpp:80

stan::gm::expression

**Definition:** ast.hpp:216

var\_decls\_grammar.hpp

stan::gm::statement\_grammar::var\_map\_

variable\_map & var\_map\_

**Definition:** statement\_grammar.hpp:33

stan::gm::statement\_grammar::var\_decls\_g

var\_decls\_grammar< Iterator > var\_decls\_g

**Definition:** statement\_grammar.hpp:38

stan::gm::statement\_grammar::expression\_g

expression\_grammar< Iterator > expression\_g

**Definition:** statement\_grammar.hpp:37

stan::gm::reject\_statement

**Definition:** ast.hpp:751

stan::gm::statement\_grammar::var\_lhs\_r

boost::spirit::qi::rule< Iterator, variable\_dims(var\_origin), whitespace\_grammar< Iterator > > var\_lhs\_r

**Definition:** statement\_grammar.hpp:167

ast.hpp

stan::gm::statement\_grammar::void\_return\_statement\_r

boost::spirit::qi::rule< Iterator, return\_statement(var\_origin), whitespace\_grammar< Iterator > > void\_return\_statement\_r

**Definition:** statement\_grammar.hpp:102

stan::gm::statement\_grammar::dims\_r

boost::spirit::qi::rule< Iterator, std::vector< expression >var\_origin), whitespace\_grammar< Iterator > > dims\_r

**Definition:** statement\_grammar.hpp:56

stan::gm::statement\_grammar::no\_op\_statement\_r

boost::spirit::qi::rule< Iterator, no\_op\_statement(), whitespace\_grammar< Iterator > > no\_op\_statement\_r

**Definition:** statement\_grammar.hpp:131

stan::gm::whitespace\_grammar

**Definition:** whitespace\_grammar.hpp:11

stan::gm::statement\_grammar::increment\_log\_prob\_statement\_r

boost::spirit::qi::rule< Iterator, increment\_log\_prob\_statement(bool, var\_origin), whitespace\_grammar< Iterator > > increment\_log\_prob\_statement\_r

**Definition:** statement\_grammar.hpp:68

stan::gm::statement\_grammar::assignment\_r

boost::spirit::qi::rule< Iterator, assignment(var\_origin), whitespace\_grammar< Iterator > > assignment\_r

**Definition:** statement\_grammar.hpp:45

whitespace\_grammar.hpp

stan::gm::statement\_grammar::sample\_r

boost::spirit::qi::rule< Iterator, sample(bool, var\_origin), whitespace\_grammar< Iterator > > sample\_r

**Definition:** statement\_grammar.hpp:146

stan::gm::return\_statement

**Definition:** ast.hpp:757

stan::gm::statement\_grammar::non\_lvalue\_assign\_r

boost::spirit::qi::rule< Iterator, expression(var\_origin), whitespace\_grammar< Iterator > > non\_lvalue\_assign\_r

**Definition:** statement\_grammar.hpp:50

stan::gm::statement\_grammar::opt\_dims\_r

boost::spirit::qi::rule< Iterator, std::vector< expression >var\_origin), whitespace\_grammar< Iterator > > opt\_dims\_r

**Definition:** statement\_grammar.hpp:136

stan::gm::while\_statement

**Definition:** ast.hpp:737

stan::gm::statement\_grammar::distribution\_r

boost::spirit::qi::rule< Iterator, distribution(var\_origin), whitespace\_grammar< Iterator > > distribution\_r

**Definition:** statement\_grammar.hpp:62

stan::common::sample

void sample(stan::mcmc::base\_mcmc \*sampler, int num\_warmup, int num\_samples, int num\_thin, int refresh, bool save, stan::io::mcmc\_writer< Model, SampleRecorder, DiagnosticRecorder, MessageRecorder > &writer, stan::mcmc::sample &init\_s, Model &model, RNG &base\_rng, const std::string &prefix, const std::string &suffix, std::ostream &o, StartTransitionCallback &callback)

**Definition:** sample.hpp:13

stan::gm::var\_origin

int var\_origin

**Definition:** ast.hpp:411

stan::gm::statement\_grammar::return\_statement\_r

boost::spirit::qi::rule< Iterator, return\_statement(var\_origin), whitespace\_grammar< Iterator > > return\_statement\_r

**Definition:** statement\_grammar.hpp:97

stan::gm::statement\_grammar::statement\_grammar

statement\_grammar(variable\_map &var\_map, std::stringstream &error\_msgs)

**Definition:** statement\_grammar\_def.hpp:527


---

[ Stan Home Page ]

*© 2011–2014,
Stan Development Team.*
